# Editorial: Nitro Compounds as Versatile Building Blocks for the Synthesis of Pharmaceutically Relevant Substances

**DOI:** 10.3389/fchem.2020.595246

**Published:** 2020-10-19

**Authors:** Alexey Yu. Sukhorukov

**Affiliations:** ^1^Laboratory of Organic and Metal-Organic Nitrogen-Oxygen Systems, N. D. Zelinsky Institute of Organic Chemistry, Moscow, Russia; ^2^Department of Innovational Materials and Technologies Chemistry, Plekhanov Russian University of Economics, Moscow, Russia

**Keywords:** nitro compounds, amines, catalysis, umpolung, nitrogen heterocycles, pharmaceuticals

In a 1979 review on the chemistry of nitro compounds, Dieter Seebach outlined that they were “ideal intermediates in organic synthesis” (Seebach et al., 1979). However, it was up to future generations to verify this statement. By the time Seeback's review was published, nitro compounds had long played important roles as intermediates in organic synthesis (e.g., reduction to amines, Henry reaction, nitro-Michael reaction, and Michael addition to nitroalkenes, etc.). Numerous data available at that time on the reactivity of the nitro group made it possible to expect the development of new synthetic methods (Ono, 2001).

After 40 years, the state-of-art in the field of nitro compounds has developed and started to be evaluated. According to Scopus, since the early 2000s, the annual number of articles that mention nitro compounds has increased by about 5 times compared to the 1980s. Most of these papers deal with the synthetic aspects of the nitro group chemistry. In the past 10 years, over 20 reviews on the chemistry and utility of nitro-derivatives in organic synthesis have been published. In 2020, two special issues dedicated to nitro compounds appeared in chemistry journals (Nishiwaki, 2020), one of which is brought to attention here. These studies demonstrate the on-going interests of organic chemists, who continue to explore nitro-derivatives as useful building blocks.

The Frontiers Research Topic “Nitro Compounds as Versatile Building Blocks for the Synthesis of Pharmaceutically Relevant Substances” comprises a collection of original research and review articles dealing with the synthesis, chemistry, and applications of nitro-derivatives. This special issue consists of six articles, which reflect on modern trends in the synthetic chemistry of the nitro group.

Reduction to the amino group, which is the oldest known reaction of the NO_2_-unit, has gained intensive development in recent years as a key stage in the one-pot synthesis of secondary and tertiary amines (so-called reductive alkylation of nitro compounds). The use of nitroalkanes and nitroarenes as precursors of primary amines in multi-stage one-pot processes is a promising strategy in sustainable pharmaceutical-oriented organic synthesis. An important challenge in this methodology is the search for efficient hydrogenation catalysts based on non-precious metals. More information about the latest advances in this field can be found in the mini-review by Sukhorukov.

Nitroalkanes and nitroalkenes are among the most widely used substrates in enantioselective organocatalytic carbon–carbon and carbon–heteroatom bond-forming reactions. The products of these transformations are optically pure functionalized nitro-derivatives, which can serve as versatile building blocks in the assembly of stereochemically complex bioactive frameworks. This is a relatively new and rapidly developing field, which is reviewed every few years (Noble and Anderson, 2013; Faisca Phillips, 2016; Sukhorukov et al., 2016). A review by Faisca Phillips et al. is focused on the application of the stereoselective nitro-Mannich reaction in the synthesis of active pharmaceutical ingredients, natural products, and other bioactive substances. This comprehensive review provides insights about progress in this area over the past 4 years. Another report by Hajra et al. explores stereoselective synthetic methods utilizing nitro compounds in Michael reaction. It describes the first catalytic and enantioselective conjugate addition of nitromethane to benzylidene-2-benzoyl acetate catalyzed by a squaramide-modified dihydroquinine organocatalyst. The study demonstrated the applicability of their protocol to the asymmetric total synthesis of a potent endothelin receptor antagonist ABT-627 (Atrasentan).

The multi-component synthesis of nitrogen heterocycles from nitroalkenes and nitroalkanes is a well-recognized strategy in heterocyclic chemistry (Halimehjani et al., 2014a,b). Two research articles dealing with this field are included in this Research Topic. A paper by Wang et al. reports a novel four-component one-pot synthesis of polysubstituted 2,3-dihydro-4-nitropyrroles from α-ketoamides, amines, aromatic aldehydes, and β-nitroalkenes or β-pivaloxy-nitroalkanes. Since pyrrole is a privileged scaffold in medicinal chemistry, this transformation may have utility in the synthesis of pharmaceutically relevant molecules. In another report, Vroemans et al. describes the preparation of unusual 5,5′-*C,C*-linked bis-1,2,3-triazoles with various substitution patterns exploiting three-component reactions between 5-formyl-1,2,3-triazole, nitroalkanes, and organic azides.

Conventional carbon–carbon and carbon–heteroatom bond forming reactions involving nitroaliphatic compounds proceed under base catalysis via nitronate anions. The latter serve as strong α-C-nucleophiles in these transformations. On the other hand, upon activation with Lewis acids reactions of *aci*-nitro tautomers can be realized. Under these conditions, *aci*-forms can exhibit umpolung reactivity (with respect to nitronate anions) and serve as strong α-C-electrophiles (Tabolin et al., 2018). An early example of this process is the well-known Nef reaction, in which the nucleophilic addition of water to the protonated *aci*-forms is the key step (Ballini and Petrini, 2004). Recently, it has been demonstrated that upon activation with certain Lewis and Brønsted acids C-, N-, and S-nucleophiles, can be introduced to the α-position of nitroalkanes and nitroalkenes leading to functionalized oximes. These transformations substantially broaden the scope in the reactivity of nitro compounds and present new applications in organic synthesis. Thus, the development of new synthetic methods exploiting the umpolung reactivity of nitroaliphatic derivatives can be anticipated in the near future. Another comprehensive review by Aksenov et al. also deals with the Lewis acid activation of the nitro-fragment.

There are three major advantages to using nitro compounds as key intermediates in the synthesis of active pharmaceutical ingredients. First, the diverse reactivity of the nitro-functionality can be used in carbon-carbon and carbon-heteroatom bond forming reactions and functional group transformations. The second advantage is that modern strategies such as asymmetric organocatalysis and transition metal catalysis have demonstrated excellent applicability to nitro compounds. Accordingly, many of their transformations can be carried out in an asymmetric variant providing access to enantiopure precursors of bioactive products. Finally, nitro-derivatives of both aliphatic and aromatic series are easily accessible through multiple synthetic routes. These advantages make nitro compounds, if not ideal, at least convenient and versatile reagents, without which it is difficult to envision modern organic synthesis. At the same time, the chemistry of the nitro group, like 40 years ago, remains an exciting and challenging research area, in which many more discoveries will be made.

## Author Contributions

The author confirms being the sole contributor of this work and has approved it for publication.

## Conflict of Interest

The author declares that the research was conducted in the absence of any commercial or financial relationships that could be construed as a potential conflict of interest.
